# Integrating environmental sustainability into the teaching of global health ethics from a students’ perspective: new guiding questions

**DOI:** 10.1080/16549716.2024.2448896

**Published:** 2025-02-03

**Authors:** Marc Sánchez Benito, Julia Bielik, Carmen Rauh Garrido, Johanna Krüger, Selina Noe, Saskia-Linda Stämmler, Salome Steinke, Sofia Tzirita, Xuan Zhang

**Affiliations:** Department of Global Public Health, Karolinska Institutet, Stockholm, Sweden

**Keywords:** Planetary health, ethical tensions, sustainable development, healthcare integration, interconnectedness

## Abstract

Environmental sustainability stands out as a crucial topic in global health. Although this concept has been part of ethical discussions for over two decades, progress toward its adoption within global health ethics education remains slow and hesitant, hindering its integration into global health practice and decision-making processes. As recent global health graduates, we believe that global health relies on the interconnectedness of human and environmental health. Therefore, we sought to address a gap in our ethics formation by proposing the inclusion of an environmental sustainability perspective in global health ethics teaching through a revised question checklist and classroom activity. This question checklist expands on the seven teaching principles for public health ethics by Schröder-Bäck et al. applying an environmental ethics lens to global health ethics. The group activity offers a structured method for students to explore practical applications of environmental sustainability in global health processes. We aim to foster more critical reflections and discussions on this topic among future global health professionals and students, paving the way for a more environmentally sustainable future in global health.

## Paper Context

- **Main findings:** There is potential for improvement in the integration of environmental sustainability into global health educational ethical frameworks.
- **Added knowledge:** Including an environmental sustainability perspective in teaching of global health ethics could be an innovative and effective way to incorporate it into future global health practices.
- **Global health impact for policy and action:** This approach can spark critical discussions and reflections among future global health professionals, ensuring environmental accountability in global health practices and endorsing sustainable transformation and reform in this field.

## Background

As recent global health graduates, we have learned about the vital importance of sustainability to ensure health. We view health holistically [1] as ‘the state of complete physical, mental and social well-being and not merely the absence of disease or infirmity’ [2] and as caring for the well-being of both current and future generations [3]. Guided by the Sustainable Development Goals (SDGs), we recognise the importance and interconnectedness of the three fundamental pillars of sustainable development: social, economic, and environmental [4].

Even in the early stages of our global health careers, we often come across reports detailing the surpassing of planetary boundaries [5] and the detrimental effects thereof on health [6]. Despite this, hesitant progress is being made towards meeting the objective of environmental sustainability within the SDGs [7]. As emerging global health professionals, our strong sense of responsibility led us to focus on the environmental dimension of sustainable development in regard to SDG 3 (Good health and well-being) while approaching sustainable health as a multisectoral effort to improve health for all within planetary limits – key to achieving the 2030 Agenda and enabling a healthy, fulfilling life for everyone [3].

Drawing from our learning experiences, we have noticed a misalignment between the recognised need for environmental sustainability and its current integration within global health ethics. Therefore, in this article, we propose a way to address this gap by incorporating an environmental sustainability perspective into global health ethics through education. Our aim is to instil a sense of environmental sustainability in ethics among emerging global health professionals, thereby shaping the future landscape of global health practices and decision-making processes.

## Approaching environmental and global health ethics

Environmental ethics examines the intersection between humans, non-human beings, and the natural environment. According to Lee, there are two separate approaches: the preservationist/deep ecology philosophy views nature as having intrinsic value and advocates for human respect towards ecosystems without exploitation, whereas the conservationist/shallow ecology perspective prioritises managing nature for human benefit, focusing on sustainable resource use to support human development and well-being [8]. Sustainability is a complex concept that has grown out of environmental ethics as it raises important issues of social equity, justice, and intergenerational impact, also reflected in its inclusion as one of the seven environmental health ethical principles, according to Resnik [9]. More specifically, environmental sustainability encompasses the conscientious utilisation of resources and the protection of our natural milieu for the future [10]. This practice is characterised by implementing conservation strategies and sustainable methodologies to foster ecosystem resilience and promote the enduring welfare of both current and future generations [10].

Global health ethics, for us, was first introduced with the exploration of the four ethical principles: non-maleficence, beneficence, respect for autonomy, and justice [11]. Subsequently, we were acquainted with the pedagogical framework by Schröder-Bäck et al. for teaching the seven principles of public health ethics, which also includes the principles of health maximisation, efficiency, and proportionality [11,12]. At the same time, through our studies, we were introduced to the concept of interconnectedness between human and animal health, as well as the environment, shifting away from the anthropocentric view of Western medicine and making space for synergies between more biocentric and ecocentric approaches [13]. This allowed us to have a more holistic approach, viewing humans as part of nature, something often reflected in the practices of indigenous populations [13]. Through all these learning experiences, we reflected extensively on applying these seven principles within current global health teaching and noticed the broad lack of consideration of environmental sustainability. However, we acknowledge that several dilemmas must be addressed when balancing environmental sustainability and a commitment to global health, encapsulated in three main ethical tensions proposed by Jameton and Pierce [14].

Firstly, ‘The Individual versus the Whole’ highlights the ethical tension between the traditional Hippocratic principle of ‘do no harm’ to the individual patient and the broader responsibility to avoid harm to both humans and nature. The ‘whole’ in this context refers to the collective well-being of society and the ecosystem. The dilemma arises when avoiding harm to the individual may result in harm to others, including future generations and the environment. It further raises the question of how these individual commitments can conflict with the need for sustainable practices that protect both the planet and future generations [14].

The second dilemma is ‘Sustainability versus Social Justice.’ Here, the complex relationship between environmental sustainability in healthcare while ensuring social justice is explored, acknowledging the difficulty of simultaneously pursuing these two potentially conflicting aims [15]. The challenge lies in ensuring high standards in healthcare without contributing to or worsening global inequalities and environmental degradation.

Finally, the tension of ‘Sustainability versus Health’ addresses the dilemma of how to maintain or improve health outcomes within the constraints of environmental sustainability [14]. Health should indeed encompass sustainability, as a healthy population depends on a stable environment. However, the historic pursuit of health improvements has often involved resource-intensive practices that harm ecological systems [16]. This dilemma highlights the need to rethink our approach to healthcare, ensuring that efforts to enhance individual well-being do not come at the expense of the environment.

These considerations are equally essential within the field of global health, which extends beyond addressing the needs of individual persons to encompass the well-being of interconnected communities across various geographies and borders, sharing resources like air, water, and land [17]. Thus, we believe that global health emerges as a valuable tool for harmonising the seemingly disparate dimensions of health [8], as it can serve as a bridge between bioethics, which concerns individual health, and environmental ethics, which focuses on environmental factors affecting health [8].

## New guiding questions: integrating environmental sustainability into the teaching of global health ethics

Expanding on the insights we gained through the global health ethics module, we identified a need to integrate environmental sustainability considerations specifically into global health ethics teaching [18]. Embedding environmental ethics into global health courses could serve as a pathway to stimulate essential discussions among future global health professionals on the interconnectedness between environmental sustainability and global health [19].

**Figure 1.**
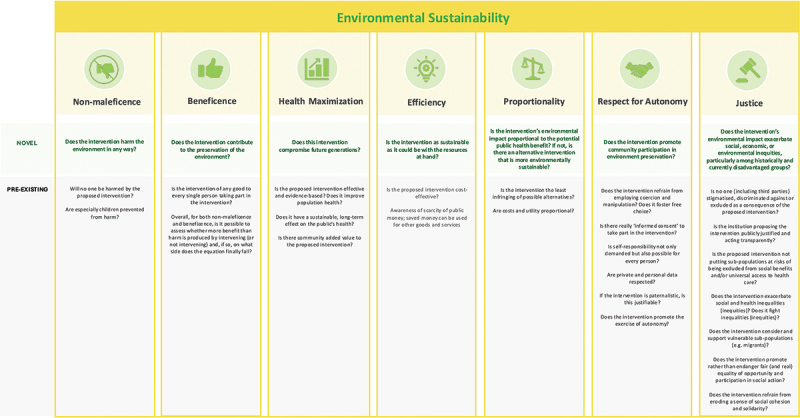
Environmental sustainability in global health ethics: proposed expansion of the seven public health teaching principles checklist from Schröder-bäck et al. [12] to include novel guiding questions (dark green) that integrate an environmental sustainability perspective.

The importance of approaching these discussions among global health students from a student-centred approach has been highlighted previously [3,12,20,21]. To support future student-led reflections on how environmental sustainability could be integral to global health ethics teaching, we introduce an environmental sustainability perspective to the seven ethical principles by Schröder-Bäck et al., which include non-maleficence, beneficence, health maximisation, efficiency, respect for autonomy, justice and proportionality [12]. To that end, we suggest adding novel guiding questions to each of these principles (marked with green in Figure 1) to address environmental sustainability in global health. We hope that this expanded global health ethics checklist, outlined in Figure 1, serves as a valuable starting point for deeper critical reflections on how to shape future global health practices toward greater environmental sustainability.

We propose that educators of upcoming global health students use this expanded checklist when teaching global health ethics. The learning objective would be to thoroughly examine current ethical gaps concerning environmental sustainability within global health practices and to propose novel approaches to address these gaps in current and future global health practices. A practical example of a student-centred activity with this expanded checklist in global health ethics classes and its use through five structured steps are described in Table 1. Students would first be divided into small groups and asked to briefly reflect on the seven ethical principles and the addition of an environmental sustainability lens. Then, each group examines a specific global health target or intervention, utilising the expanded checklist. Following that, students identify potential gaps and barriers in addressing sustainability within their chosen intervention and propose solutions. Finally, each group presents their findings to the class, sparking a broader exchange of ideas. This will allow students to recognise the complexity of balancing sustainability with other ethical obligations in global health.

**Table 1.** Student-centred activity example: reflecting on the integration of the ethical perspective of environmental sustainability.

Step	Group Activity
(1)	Divide students into small groups.
(2)	Discuss and critically reflect on the ethical perspective of environmental sustainability within the seven ethical principles by Schröder-Bäck et al. [12].
(3)	Discuss the ethical responsibilities within one current global health intervention by using the proposed expanded checklist focusing on environmental sustainability (Table 1). Target example: Healthcare supply chains are associated with heavy carbon emissions, as supplies are often produced far from where they are used, creating a significant carbon footprint due to transportation and production emissions. Example of how new guiding questions could be used and answered in regards to the target: Non-maleficence: Does the intervention harm the environment in any way? Yes, the healthcare supply chain’s reliance on long-distance transportation and production often generates significant carbon emissions, harming the environment through pollution and climate change. Health maximisation: Does the intervention compromise future generations? By contributing to climate change, these carbon emissions create long-term environmental and health risks that will disproportionately affect future generations. Efficiency: Is the intervention as suitable as it could be with the resources at hand? No, more sustainable alternatives, such as local sourcing and renewable energy use, could reduce environmental impact while maintaining healthcare supply efficiency.
(4)	Identify potential gaps or barriers in addressing environmental sustainability within the global health intervention and more thoroughly reflect on how these could be addressed in future global health practices. e.g. In the supply chain example, groups might highlight the lack of accountability for emissions, which could be mitigated by sourcing local materials or implementing more sustainable transport options.
(5)	Present and discuss findings with the whole class.

## Key considerations for integrating environmental sustainability into global health ethics education

Future discussion and implementation of the ethical perspective of environmental sustainability necessitates a reflective analysis of the tension between ethical responsibility towards both humans and the environment within global health practices. Similarly, the question of whose interest and welfare we prioritise concerning environmental sustainability, whether human, environmental, or planetary health, was at the heart of our debate. Due to three main reasons, we decided to focus the checklist’s ethical perspective and guiding questions entirely on the ethical responsibility towards the environment.

Firstly, environmental and human health are inextricably interlinked [21]. Thus, directly or indirectly, aiming to achieve positive outcomes for human and environmental health is an overlapping cause. Secondly, the seven ethical principles by Schröder-Bäck et al., which we use as a launching pad for our perspective, centre around community and population health, leaving a specific need to consider environmental interests [12]. Thirdly, we found that promoting sustainability as a guiding perspective and active practice for global health was a novel highlight missing from bioethics or mainstream public health ethics teaching [22]. We observed our wrestling between competing interests of humans and the environment turning into a fruitful opportunity to seek out common benefits, opportunities for innovation, and means of responsible practice, resulting in a change from binary thinking towards embracing mutuality: respecting both humans AND the environment.

Additional considerations in the development process of the new guiding questions arose during the integration of environmental sustainability into three of the existing ethical principles – autonomy, health maximisation, and efficiency. Firstly, with regard to autonomy, we discussed how respect for individual agency could be applied to environmental sustainability within health and particularly wanted to highlight the importance of promoting community participation in environmental preservation [11]. In the context of health maximisation, which prioritises the greater benefit of many over the few, we expanded our focus to consider the intergenerational impact of interventions on both current and future generations [12]. It is essential that global health practices are designed to ensure that their outcomes benefit and do not harm future generations, as well as provide a sustainable use of resources to promote long-term well-being and ecological balance. Lastly, our discussion focused on the tension between efficiency and sustainability, where prioritising cost-effectiveness can lead to unsustainable practices and investing in sustainable designs may not always optimise resource efficiency. Therefore, we urge students to reflect on how limited natural resources can be managed and used effectively, shifting the emphasis from cost-effectiveness towards responsible resource management to strive for both maximum environmental sustainability and efficiency.

Lastly, it is imperative to acknowledge that the implementation of these guiding questions for environmental sustainability in global health ethics teaching will vary across diverse contexts, each characterised by its own specific needs and challenges. Ultimately, a more expansive and inclusive framework for global health ethics may emerge, recognising the profound interconnectedness between humans, the environment, and all forms of life on Earth.

## Conclusion

Introducing environmental sustainability as a core ethical perspective in global health teaching could offer an innovative solution to integrate environmental sustainability into global health education, making it more tailored to contemporary global health and sustainability challenges. These supplementary guiding questions act as a normative global health ethical framework, aiming to foster critical thinking and ignite fruitful discussions and reflections. Through our proposal, which acknowledges the need for humans to assume an integrated, proactive, and even positive role in environmental preservation, we hope to foster a commitment to environmental sustainability within global health education beyond the classroom among current and future generations.
